# Prevalence of bruxism in children with episodic migraine - a case–control study with polysomnography

**DOI:** 10.1186/1756-0500-7-298

**Published:** 2014-05-14

**Authors:** Alice Hatsue Masuko, Thais Rodrigues Villa, Marcia Pradella-Hallinan, Alexander Joseph Moszczynski, Deusvenir de Souza Carvalho, Sergio Tufik, Gilmar Fernandes do Prado, Fernando Morgadinho Santos Coelho

**Affiliations:** 1 Disciplina de Neurologia, Departamento de Neurologia e Neurocirurgia e da Universidade Federal de São Paulo, São Paulo, Brazil; 2 Disciplina de Medicina e Biologia do Sono, Departamento de Psicobiologia da Universidade Federal de São Paulo, São Paulo, Brazil; 3 Robarts Research Institute, Western University, London, Ontario, Canada; 4 Rua Marselhesa, 529, Vila Clementino, CEP 04020-060, São Paulo, SP, Brasil

**Keywords:** Bruxism, Sleep, Migraine, Polysomnography

## Abstract

**Background:**

Parents of children with migraine have described a higher prevalence of sleep bruxism and other sleep disturbances in their children. The objective of this study was to use polysomnography to investigate the prevalence of bruxism during sleep in children with episodic migraine relative to controls.

**Findings:**

Controls and patients were matched by sex, age, years of formal education, presence of snoring, arousals per hour, and respiratory events per hour.

A total of 20 controls, between 6 and 12 years old, with no history of headache, recruited from public schools in Sao Paulo between 2009 and 2012, and 20 patients with episodic migraine recruited from the Headache Clinic at the Federal University of Sao Paulo between 2009 and 2012 underwent polysomnography.

No intervention was performed before sleep studies.

Among migraine patients, 27.5% experienced aura prior to migraine onset. The sleep efficiency, sleep latency, REM sleep latency, arousals per hour, percentage of sleep stages, and breathing events per hour were similar between groups. Five children (25%) with episodic migraine exhibited bruxism during the sleep study while this finding was not observed in any control (p = 0.045).

**Conclusions:**

Our data demonstrate that bruxism during sleep is more prevalent in children with episodic migraine. Further prospective studies will help elucidate the underlying shared pathogenesis between bruxism and episodic migraine in children.

## Findings

About seventy percent of children experience at least one episode of any kind of headache [1-3]. Headache has been described as a comorbidity with sleep disorders, and it is thought that the two share a common pathophysiological pathway, however this relationship is currently not well understood [4].

In a healthy pediatric population the prevalence of sleep bruxism is about 18% with risk of tooth damage and pain [5,6]. Sleep bruxism is associated with stress, anxiety, blood pressure fluctuations, cortical arousals and body movements with consequent sleep fragmentation [7].

One polysomnographic study has previously reported an increased prevalence of sleep bruxism in tensional headache patients [8]. Likewise, parents of children with migraine have described a higher than normal prevalence of sleep bruxism and other sleep disturbances [9]. The objective of this study was to describe the prevalence of bruxism during sleep in children with episodic migraine relative to controls.

This study was approved by the Ethics Committee at the Federal University of São Paulo – Unifesp, under record number 1522/09. All participants’ parents signed informed consent.

A total of 20 controls, between 6 and 12 years old, with no history of headache, no regular medication use and no neurological symptoms were recruited from public schools in Sao Paulo between 2009 and 2012. A neurologist examined all children before enrollment and a headache diary was collected for 8 weeks.

20 patients with episodic migraine, as defined by the International Headache Society (ICHD II, 2004), between 6 and 12 years old, were recruited from the Headache Clinic at the Federal University of Sao Paulo between 2009 and 2012. No patient used any regular medication or had more than 4 attacks per month.

Variable normality was verified using the Kolmogorov–Smirnov Test. The Chi-square test and the Fisher’s exact test were used to analyze qualitative variables. The Student’s t test for independent samples and the Mann–Whitney U test were used to compare quantitative variables. Statistical significance was set to p < 0.05.

The groups were matched by sex, age, years of formal education, presence of snoring, arousals per hour, and breathing events per hour. All participants underwent an overnight sleep study (Table 1). Standard polysomnographic recordings were obtained using the Alice 3 system. Sleep stages were analyzed according to the Rechtschaffen and Kales criteria, which was the standard at the time the studies were scored. Respiratory events were evaluated according to the recommendations of the 1999 Task Force of the American Academy of Sleep Medicine. Arousals and periodic limb movements were analyzed according to ASDA criteria [10-12]. Bruxism was scored according to the AASM 2007 criteria [13].

**Table 1:** Clinical and sleep characteristics of migraine patients and controls

	**Healthy children**	**Migraine patients**	**p**
Sex (male)	10	10	1
Age	9.05 ± 1.66	9.5 ± 0.48	0.85
Years of formal education	3.5 ± 1.5	3.9 ± 1.8	0.75
Migraine episodes per month before treatment	0	2.15 ± 1.8	0.001
Apnea and hypopnea index	0.46 ± 0.40	0.21 ± 0.20	0.10
Sleep efficiency	77.85 ± 13.85	79.94 ± 12.24	0.05
Sleep latency	49 ± 37.04	47.87 ± 41.75	0.10
REM sleep latency	141.70 ± 53.9	154.33 ± 54.6	0.10
Arousals per hour	7.09 ± 3.22	8.46 ± 3.20	0.71
N1%	5.19 ± 2.65	2.65 ± 2.24	0.18
N2%	44.29 ± 8.59	47.42 ± 7.8	0.94
N3%	32.44 ± 10.09	32.91 ± 8.6	0.10
REM%	18.06 ± 3.78	17.08 ± 6.52	0.06
Snoring	18	20	0.90
Bruxism	0	5	0.045
Epileptifom activity	2	5	0.17
Saturation of oxygen	97.06 ± 1.06	95.6 ± 1.84	0.15
Awake - Heart beat	85.10 ± 7.73	92.27 ± 24.83	0.10
NREM - Heart beat	75.80 ± 8.54	90.23 ± 39.4	0.05
REM - Heart beat	78.23 ± 24.52	83.92 ± 35.34	0.10

Within the migraine group, 27.5% experienced migraines accompanied by auras. Sleep efficiency, sleep latency, REM sleep latency, arousals per hour, percentage of sleep stages, and breathing events per hours were similar between groups (Table 1).

Five children (25%) with sporadic migraine exhibited bruxism during sleep studies, while no control subject experienced this phenomenon (p = 0.045).

None of the subjects exhibiting sleep bruxism had migraines accompanied by aura. One patient had sleep bruxism and epileptiform activity simultaneously. All patients and controls had a normal number of respiratory events per hour.

## Discussion

Although this study is limited by sample size, possible referral bias, and variability of bruxism from night to night, our data have demonstrated that sleep bruxism is more prevalent in children with episodic migraine than in healthy controls. This is in keeping with previous studies showing a higher prevalence of sleep bruxism in tensional headache patients [8]. However, no previous study has shown this trend using sleep studies to compare bruxism during sleep in a migraine group compared to healthy controls.

Migraine and sleep bruxism are periodic paroxysmal disorders with associated comorbidity and symptoms. They may share various pathophysiological mechanisms, but this relationship is currently not understood. Although sleep bruxism and migraine may both be considered autonomic dysfunctions, it is uncommon to encounter children with disorders of autonomic etiology.

Stress and consequent sympathetic activation is considered to be an important trigger for migraine and sleep bruxism [14]. Sleep bruxism is largely considered to be an autonomic dysregulation accompanied by tachycardia and microarousals. Clonidine, a sympathoylitic medication is able to reduce the sympathetic cardiac activation accompanying bruxism episodes during sleep [6]. Interestingly, migraines are thought to be the result of chronic sympathetic dysregulation as well [14]. Pharmacologically, the two disorders can be tied together by the use of propranolol, a beta-adrenergic antagonist that decreases masseter muscle contraction during sleep and is also a commonly used prophylactic drug for the treatment of migraines.^15^ This similar treatment for both conditions may indicate a similar pathophysiology pointing toward one common mechanism responsible for sleep bruxism and migraine [6]. A better understanding of the role of sympathetic nervous system dysfunction as it pertains to bruxism and migraine will be important to prevent and treat these two disorders [14,15].

On the other hand, sleep bruxism and migraine are related to a disturbance of the dopaminergic system as well. Levodopa drives a significant reduction in sleep bruxism in patients with Parkinson’s disease, while patients being treated with dopaminergic blockers have an increased prevalence of bruxism during sleep [6]. In migraine there is a well documented hypoactivation of the dopamine system and the prevention of migraine attacks depends on stationary dopaminergic tone [16]. This convergence of changes to the normal function of the dopaminergic system indicates another potential etiology of both diseases. Further studies must be completed to determine the involvement of autonomic and/or dopaminergic dysfunction as common etiologies responsible for migraine or sleep bruxism.

## Competing interests

The authors declare that they have no competing interests.

## Authors’ contributions

AHM: Substantial contributions to the conception or design of the work; or the acquisition, analysis, or interpretation of data for the work; Drafting the work or revising it critically for important intellectual content; Final approval of the version to be published; Agreement to be accountable for all aspects of the work in ensuring that questions related to the accuracy or integrity of any part of the work are appropriately investigated and resolved. TRV: Substantial contributions to the conception or design of the work; or the acquisition, analysis, or interpretation of data for the work; MPH: Substantial contributions to the conception or design of the work; or the acquisition, analysis, or interpretation of data for the work; AJM: Substantial contributions to the conception or design of the work; or the acquisition, analysis, or interpretation of data for the work; DSC: Substantial contributions to the conception or design of the work; or the acquisition, analysis, or interpretation of data for the work; ST: Substantial contributions to the conception or design of the work; or the acquisition, analysis, or interpretation of data for the work; GFP: Substantial contributions to the conception or design of the work; or the acquisition, analysis, or interpretation of data for the work; FMSC: Substantial contributions to the conception or design of the work; or the acquisition, analysis, or interpretation of data for the work; Drafting the work or revising it critically for important intellectual content; Final approval of the version to be published; Agreement to be accountable for all aspects of the work in ensuring that questions related to the accuracy or integrity of any part of the work are appropriately investigated and resolved. All authors read and approved the final manuscript.
